# Optical Design of a Miniaturized 10× Periscope Zoom Lens for Smartphones

**DOI:** 10.3390/mi14061272

**Published:** 2023-06-20

**Authors:** Wen-Shing Sun, Yi-Hong Liu, Chuen-Lin Tien

**Affiliations:** 1 Department of Optics and Photonics, National Central University, Chungli 32001, Taiwan; wssun@dop.ncu.edu.tw (W.-S.S.); yihongluis@gmail.com (Y.-H.L.); 2 Department of Electrical Engineering, Feng Chia University, Taichung 40724, Taiwan

**Keywords:** optical design, periscope zoom lens, smartphone camera, aspheric lens, tolerance analysis

## Abstract

The size of the optical zoom system is important in smartphone camera design, especially as it governs the thickness of the smartphone. We present the optical design of a miniaturized 10× periscope zoom lens for smartphones. To achieve the desired level of miniaturization, the conventional zoom lens can be replaced with a periscope zoom lens. In addition to this change in the optical design, the quality of the optical glass, which also affects the performance of the lens, must be considered. With advancements in the optical glass manufacturing process, aspheric lenses are becoming more widely used. In this study, aspheric lenses are incorporated into a design for a 10× optical zoom lens with a lens thickness of less than 6.5 mm and an eight-megapixel image sensor. Furthermore, tolerance analysis is carried out to prove its manufacturability.

## 1. Introduction

Modern-day smartphones have the ability to take images as cameras. In order to improve the optical performance of smartphone lenses, the miniaturization design of high-magnification optical zoom lenses is very important. In general, the component that most affects the performance of smartphones in taking photographs is the optical lens. In order to shrink the optical system, Shen et al. [1,2] proposed a needle beam technology that could be applied to metalenses to reduce the size of optical components. However, in addition to the correction of aberration and chromatic aberration, optical design must also consider distortion and relative illumination, especially in the design of large-angle or high-magnification zoom lenses. This is the main reason why traditional lens designs cannot be replaced. With advancements in technology [3], the lens design used in smartphone cameras to improve the quality of the photographs has evolved from the original prime lens to a multi-camera digital zoom system to an optical zoom system [4]. However, due to the size limitations of the smart phone, it is difficult to install a traditional zoom lens system. There have been many optical designs developed to solve this problem, with the periscope zoom lens being one of them.

Between the years 2008 to 2013, Park et al. published three articles describing the design of 3×, 5×, and 10× periscope zoom lenses with lens depths of 7 mm, 8 mm, and 12 mm, respectively [5,6,7]. In 2016, Sun et al. designed a 3× zoom five-megapixel periscope smartphone lens with a lens depth of 6 mm [8]. In 2018, Zhao et al. described a 2× optical periscope-based zoom design [9], with a system thickness equal to 6.5 mm, which was small enough to allow the camera to be incorporated into the smartphone. In 2020, Hou et al. proposed a periscope zoom camera with Alvarez freeform elements [10]. The camera module was designed to be 25 mm (width) × 25 mm (length) × 6 mm (height) in size. Sun et al. announced the development of a 3× zoom lens system with an eight-megapixel image sensor in which the lens depth of the zoom lens was 5.377 mm [11]. Carles and Harvey took advantage of multiple rotated rectangular apertures and the folding of the optical system [12] to allow a significant reduction in depth compared to traditional telephoto lenses. Apple Inc. obtained a US patent in 2021 [13] for a periscope-type prime lens system designed using a 4.5 mm diagonal sensor, operating at F/1.6 with 36.8° full FOV, and a lens depth of 6.5 mm. The patent implies that only lens modules less than 6.5 mm in depth can be put into a smartphone. In addition to the design of an optical system that will allow a reduction in the size, progress in lens manufacturing technology [14,15] also plays a pivotal role in improving photographic performance. For example, aberration of the optical system can be corrected with the use of aspheric lenses. In the past, the manufacture of aspheric lenses was difficult and expensive, but recent progress in glass manufacturing and the maturation of precision glass molding (PGM) technology are expected to greatly improve the quality of optical lenses.

To solve the problem of the thickness of the zoom lens being limited by the smartphone space, a design method for a 10× periscope zoom lens is proposed. The study focuses on the parameters affecting camera thickness, especially for the design of 10× periscope zoom lenses with aspheric lenses. In order to reduce the depth of the zoom lens to less than 6.5 mm, the circular aperture lens is replaced by a rectangular aperture lens. Finally, the design performance is discussed and a tolerance analysis [16] is performed to evaluate the manufacturability of the lenses.

## 2. Materials and Methods

The factors that affect the lens size for the miniaturization of a high-magnification optical zoom lens for smartphone applications are discussed below.

### 2.1. Periscope Zoom Lens

**Figure 1:**
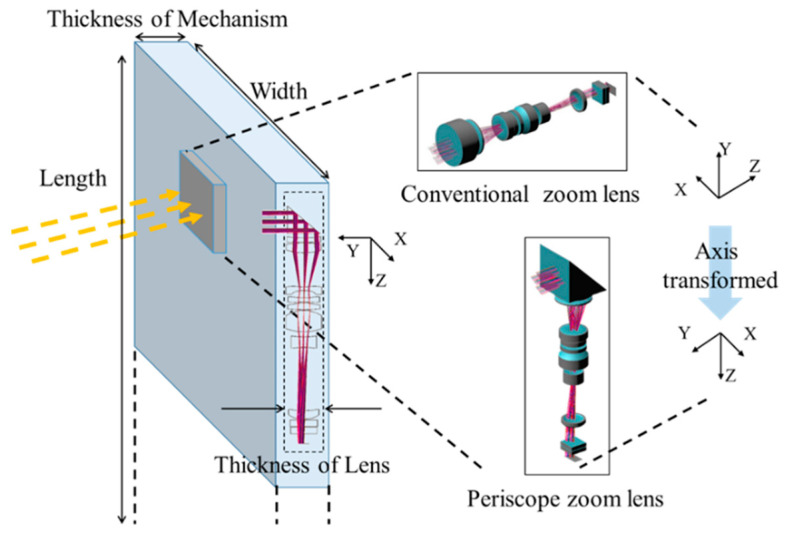
Schematic of a conventional and a periscope zoom lens for use in smartphones.

Smartphones have only a limited amount of internal space available for components other than the mobile phone, so if there is a need for a high-magnification optical zoom lens, the conventional zoom system cannot be used, because of limitations in the thickness of the phone, and a periscope zoom lens is used, as shown in Figure 1. The periscope zoom lens uses a right-angle prism to turn the original optical path by 90 degrees. The thickness of the lens is determined by the size in the Y-direction of each lens or the prism, so that the zoom system has enough space for the embedding of a high-magnification zoom lens in a smartphone.

### 2.2. Setting the Sensor Aspect Ratio

**Figure 2:**
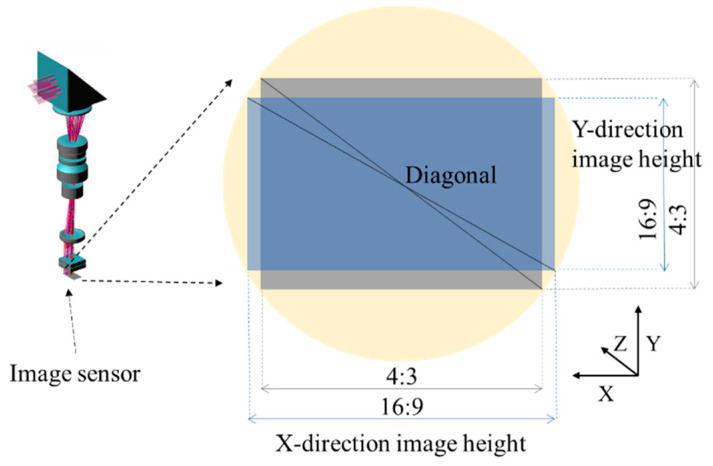
Schematic of 16:9 and 4:3 sensor aspect ratios.

The aperture size of the periscope zoom lens in the Y-direction is the most important factor affecting the thickness of the smartphone, so the selection of the image sensor before starting to design the periscope zoom lens is very important. It can be seen from Figure 2 that, under the same image height (Diagonal/2), the lens aperture size in the Y-direction is smaller for sensors with an aspect ratio of 16:9 than those with a ratio of 4:3. Therefore, using a sensor with an aspect ratio of 16:9 can effectively reduce the lens aperture size in the Y-direction, and at the same time reduce the thickness of the smartphone.

### 2.3. First-Order Lens Design

**Figure 3:**
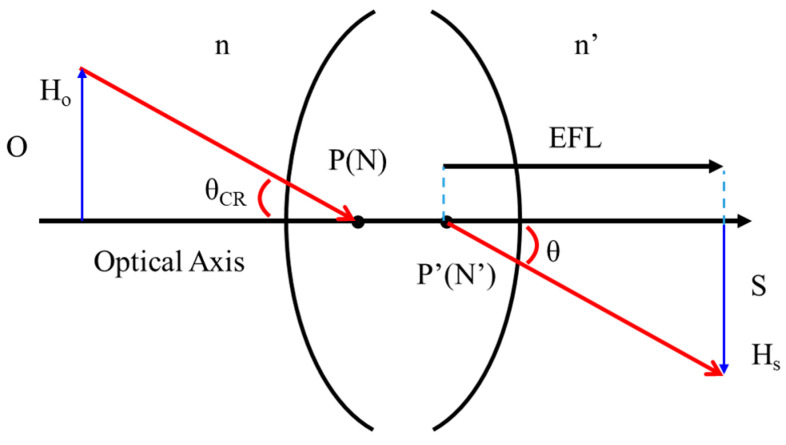
Relationship between image height, effective focal length, and half viewing angle.

Figure 3 demonstrates the half-field angle and F-number used in the optical system. Assuming that the object (O) is located at an infinite distance, the refractive index of the object is n, and the height of the object is H_o_. When the light from the object enters the optical system, an angle (θ_CR_) will be formed between the chief ray and the optical axis. When the refractive index in the object space and the image are equal (n = n′), the principal point coincides with the nodal point (namely P(N) = P′(N)). At this time, θ_CR_ = θ is equal to the half-angle field of view (HFOV), and the image height (H′) is equal to the effective focal length (EFL) times tan(θ) on the image plane (S), as expressed in Equation (1).

H_s_ = EFL × tan(θ) (1)

In this optical system, the F-number (*f*/#) refers to the ratio of the system’s focal length (EFL) to the diameter of the entrance pupil (D_en_), as shown in Equation (2). The lens depth is related to the zoom lens’ half-field angle, F-number, entrance pupil diameter, and image height. After selecting the image sensor, the image height (H_s_) will be decided. The lens depth can be reduced by decreasing the half viewing angle, increasing the F-number, reducing the diameter of the entrance pupil, or adjusting the position of the entrance pupil, as shown in Equation (3).

F-number = EFL/D_en_ (2)

H_s_ = F-number × D_en_ × tan(θ) (3)

### 2.4. Production of an Aspheric Lens through the Precision Glass Molding (PGM) Process

Aspheric lenses are better able to correct aberrations, and fewer elements are required than in spherical lens systems, which reduces system size and potentially the overall cost of production. Due to their improvement in image quality over traditional spherical optics, aspheric lenses are becoming increasingly more important in every aspect of the optics and imaging industries. The “aspheric surface” in the optical design is an optical element surface described by a high-order polynomial equation. This surface can be axisymmetric, off-axis, or non-rotationally symmetric.

The surface profile (sag) of an aspheric lens can be defined by the following formula.

(4) $$Z\left(r\right)=\frac{Cr^{2}}{1+\sqrt{1-\left(1+k\right)Cr^{2}}}+A_{4}r^{4}+A_{6}r^{6}+A_{8}r^{8}$$

where Z is the sag of the surface parallel to the optical axis; R is the radial distance from the optical axis; C is the curvature, the inverse of the radius; K is the conic constant; and A4, A6, A8, and so on indicate the fourth-, sixth-, and eighth-order aspheric coefficients, respectively.

The traditional process for fabricating optical glass lenses, which includes grinding, polishing, and lapping, is both time-consuming and expensive. Among the many fabrication techniques available, precision glass molding (PGM) can significantly reduce the time and cost required for producing optical glass, making it a promising method for aspheric and irregular glass optics technology.

PGM is a thermal molding process that involves the heating of an optical glass preform to above its glass transition temperature (T_g_), followed by the application of compression, to mechanically press the preform into a mold cavity, and then cooling and demolding the formed element. In the PGM process, optical glass with a lower T_g_ has a higher moldability. The Schott company defines glass with a transition temperature (T_g_) of less than 550 °C as moldable [17].

## 3. Design Results and Discussion

### 3.1. Lens Data for a 10× Periscope Zoom Lens

The CODE V optical design software was used to simulate, optimize, and analyze the optical systems described in this paper. The design of the 10× periscope zoom lens for use in a smartphone is described below.

**Figure 4:**
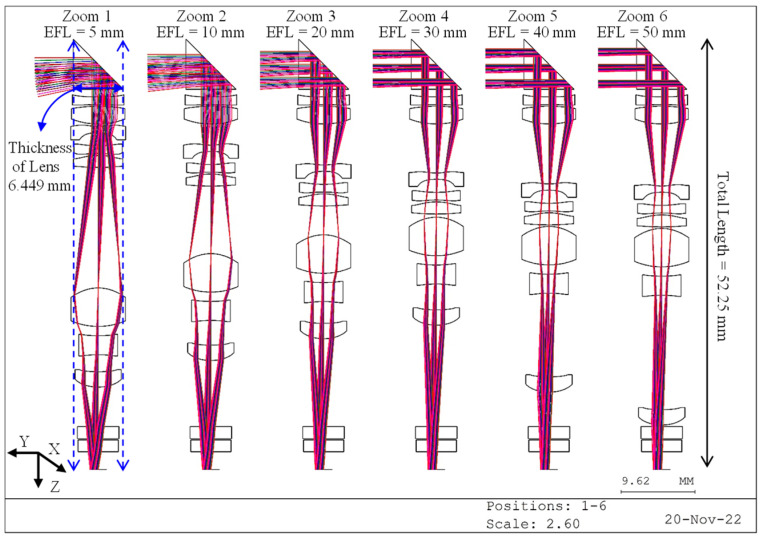
Plot of the lens configuration for the 10× optical zoom periscope lens design with an 8-megapixel image sensor.

In the CODE V optical software, the clear aperture of the lens is determined by the ray tracing of five reference rays [18]. The surface aperture of each surface is determined by the maximum ray height of five reference rays tracked by the center wavelength at different half-field angles and different focal lengths. The lens configuration is shown in Figure 4, in which we can see the optical tracking and thickness of lens of each zoom. It uses one prism, two aspheric plastic lenses, and six aspheric glass lenses with a T_g_ value of less than 550 °C. By placing a prism in front of the lens group, the optical path of the system can be turned 90 degrees, so that the lens group can zoom in the Z-direction without increasing the thickness of the optical system. In order to make the design as practical as possible for manufacture in the real world, a simulation of the design was carried out using glass from the SCHOTT company. However, during the simulation process, there was no suitable molded glass with the desired refractive index and Abbe number for the #12 and #14 aspheric surface. Finally, the EP8000 and PMMAO plastic were chosen as alternative materials, which are readily available in the market.

The lens design uses an eight-megapixel image sensor with 3840 × 2160 pixels that is produced by Omnivision: model OV16A10 (format supported). Because the pixel size is 1 μm, the image height is 2.203 mm, which can be calculated as half of the diagonal of the effective array. The effective focal length (EFL) of the design is adjustable between 5 mm (Zoom 1) and 50 mm (Zoom 6), meaning that the optical system has an optical magnification of 10. Table 1 shows the fabrication data for the 10× periscope optical zoom lens. Table 2 shows the coefficient data for the aspheric surfaces. Table 3 shows the zooming locus of the 10× periscope zoom lens. The F-numbers for the wide and telephoto angle are 3 and 12, respectively. The thickness of lens can be reduced by decreasing the diameter or adjusting the position of the entrance pupil. The diameters and positions of the entrance pupil (D_en_) at different zoom positions are given in Table 4.

The position (x, y, z) where the light passes through the lens with the largest aperture can be found using the ray-tracing method. Since the rectangular aperture size is smaller in the Y-direction than in the X-direction, a rectangular aperture can be used to remove the unnecessary part of the lens in the Y-direction, thereby allowing for a further reduction in the thickness of the camera. The circular aperture for lens surfaces #1 to #9 is replaced with a rectangular aperture lens. The maximum lens aperture size in the Y-direction is 6.311 mm, and the thickness of prism is 6.449 mm, which means that the overall thickness of the 10× periscope zoom lens can be less than 6.5 mm. The length of the lens is 52.25 mm, which is short enough to fit in a smartphone.

### 3.2. Image Performance with This Design

**Figure 5:**
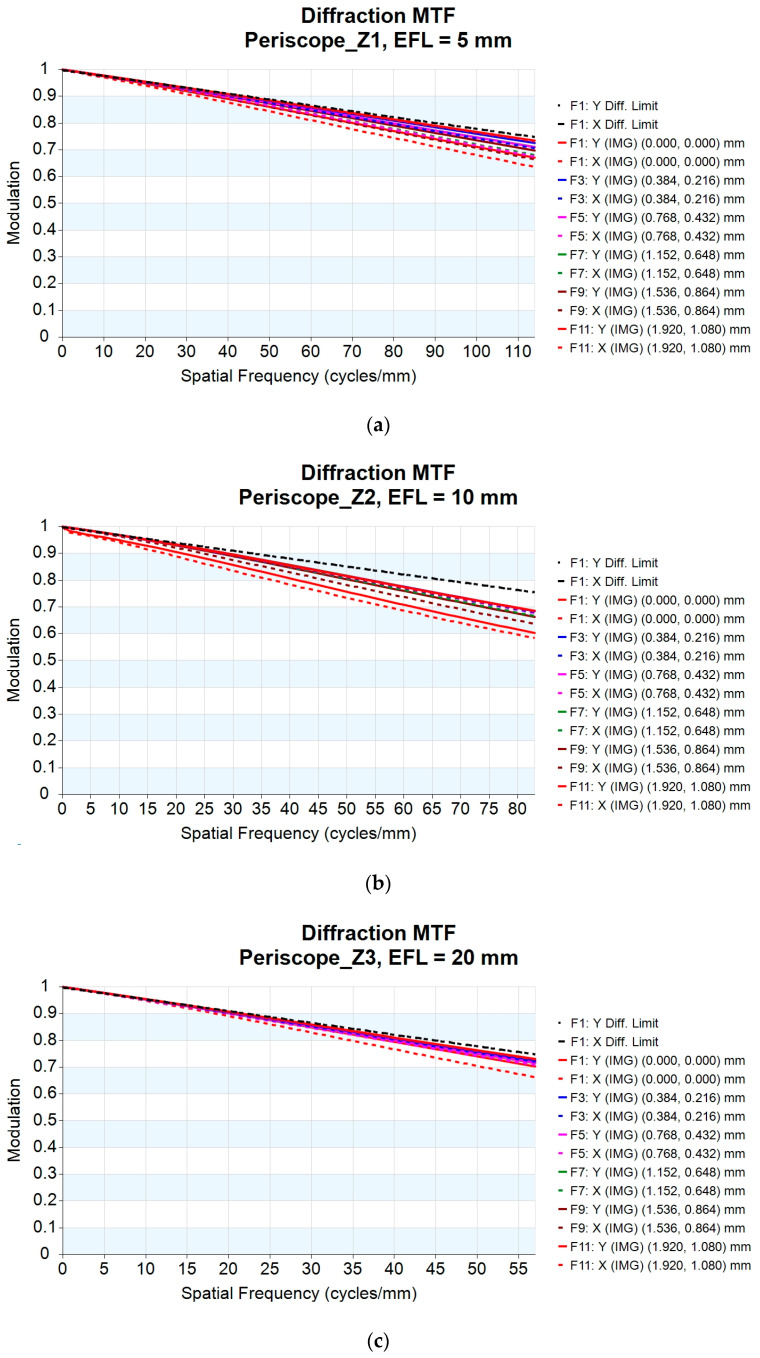

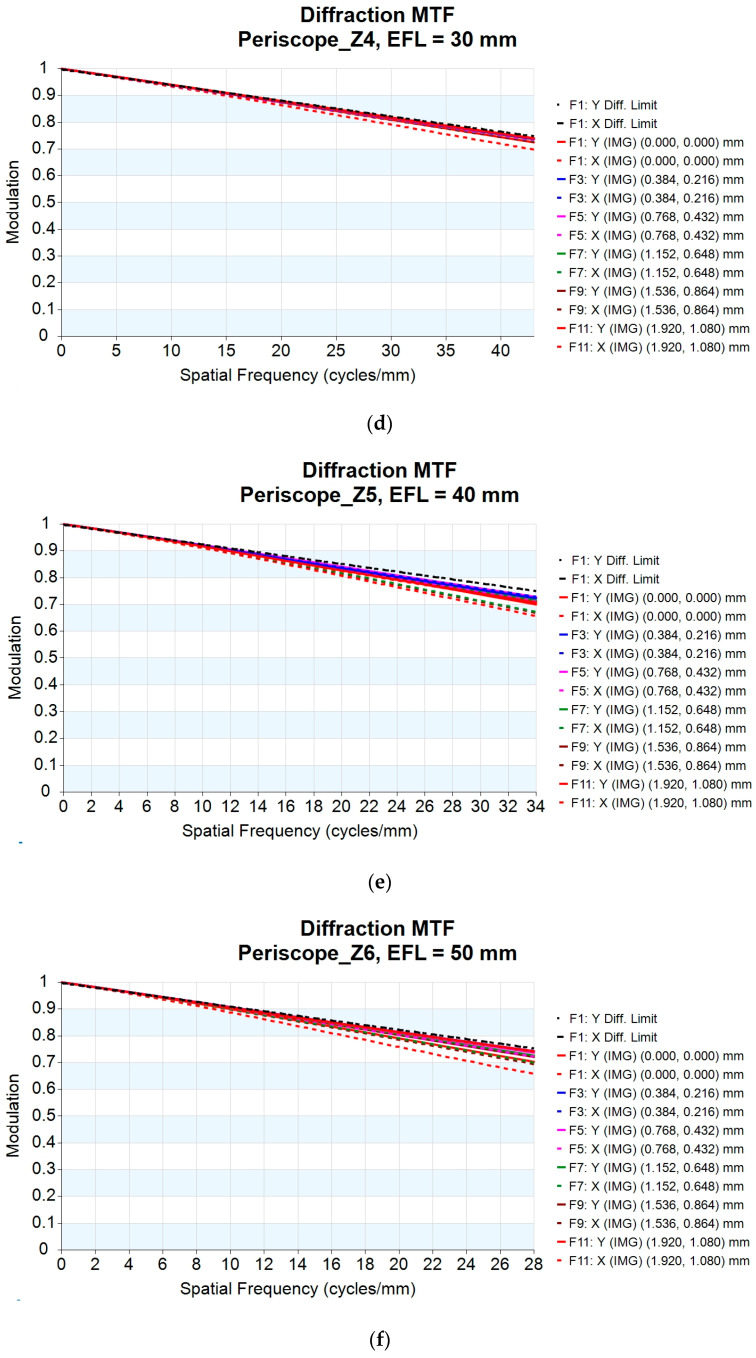
MTF plot for the 10× periscope zoom lens at (**a**) EFL = 5 mm; (**b**) EFL = 10 mm; (**c**) EFL = 20 mm; (**d**) EFL = 30 mm; (**e**) EFL = 40 mm; (**f**) EFL = 50 mm.

Considering the design performance and tolerance, the modulation transfer function (MTF) spatial frequency (freq.) is chosen to be 0.75 times the MTF diffraction limit in every field for all zoom positions. The value for the design must be greater than 0.5. Aspheric lenses are used to correct for the degradation in quality caused by spherical aberration, as well as to optimize the MTF in every field at every zoom position. The spatial frequencies for the zoom lens design are 114 lp/mm, 83 lp/mm, 57 lp/mm, 43 lp/mm, 34 lp/mm, and 28 lp/mm, where X represents the sagittal MTF curve and Y represents the tangential MTF curve. For the 10× periscope zoom lens design, the minimum MTF value is 58.60% for zoom 2 and the maximum MTF value is 74.30% for zoom 6. The MTF plots for each zoom position are shown in Figure 5a–f.

**Figure 6:**
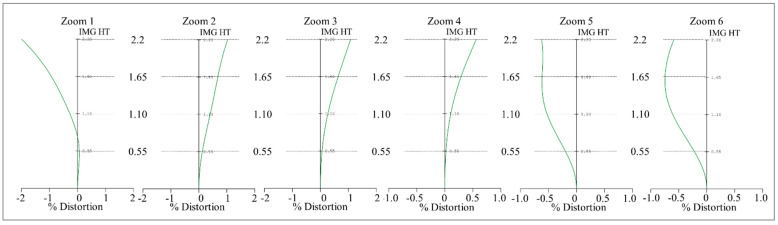
Optical distortion for the 10× periscope zoom lens from zoom 1 to zoom 6.

**Figure 7:**
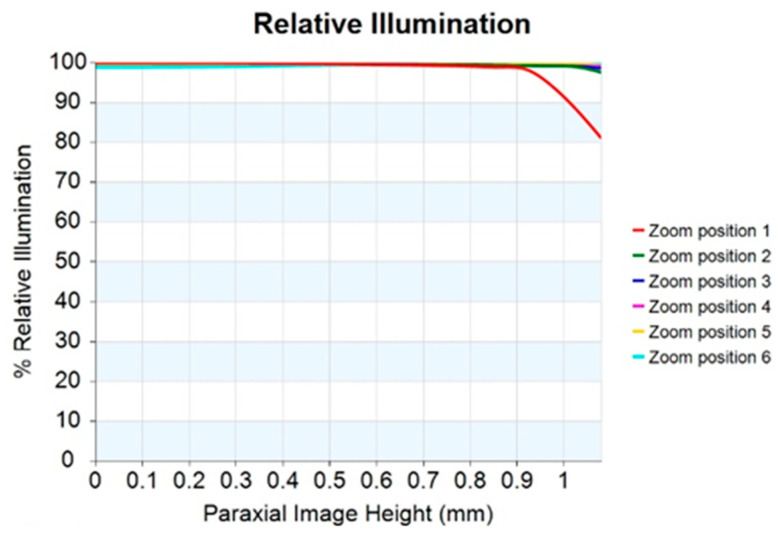
Relative illumination for the 10× periscope zoom lens design at every zoom position.

At the same time, this design can also correct optical distortion, which is one of the most important requirements for quality assessment. Optical distortion usually becomes larger with an increase in the HFOV. In this work, the optical distortion at each zoom position is below 2%, with the maximum optical distortion at zoom 1 being 2%; the optical distortion for zoom 1 to zoom 6 is shown in Figure 6. Figure 7 shows the relative illumination curves for the 10× periscope zoom lens design. The red curve indicates the minimum relative illumination of 81.07% for a zoom lens with a focal length of 5 mm.

For optical systems, the main purpose of tolerance analysis is to measure the overall effect of the combination of optical components with different tolerance ranges, so as to improve production yield and reduce costs while still meeting system performance requirements. Before performing a tolerance analysis, it is necessary to set various tolerance values for the optical components. Tolerance values vary due to differences in the processing technology of each component. The tolerance class macro (TOLCLASS.seq) included in the vender-provided macro library for CODE V [18] is used to calculate the cumulative probability with two standard deviations 2σ (97.7%). The vendor-suggested tolerances are used for analysis of this design. The tolerance parameters are defined in Table 5.

**Figure 8:**
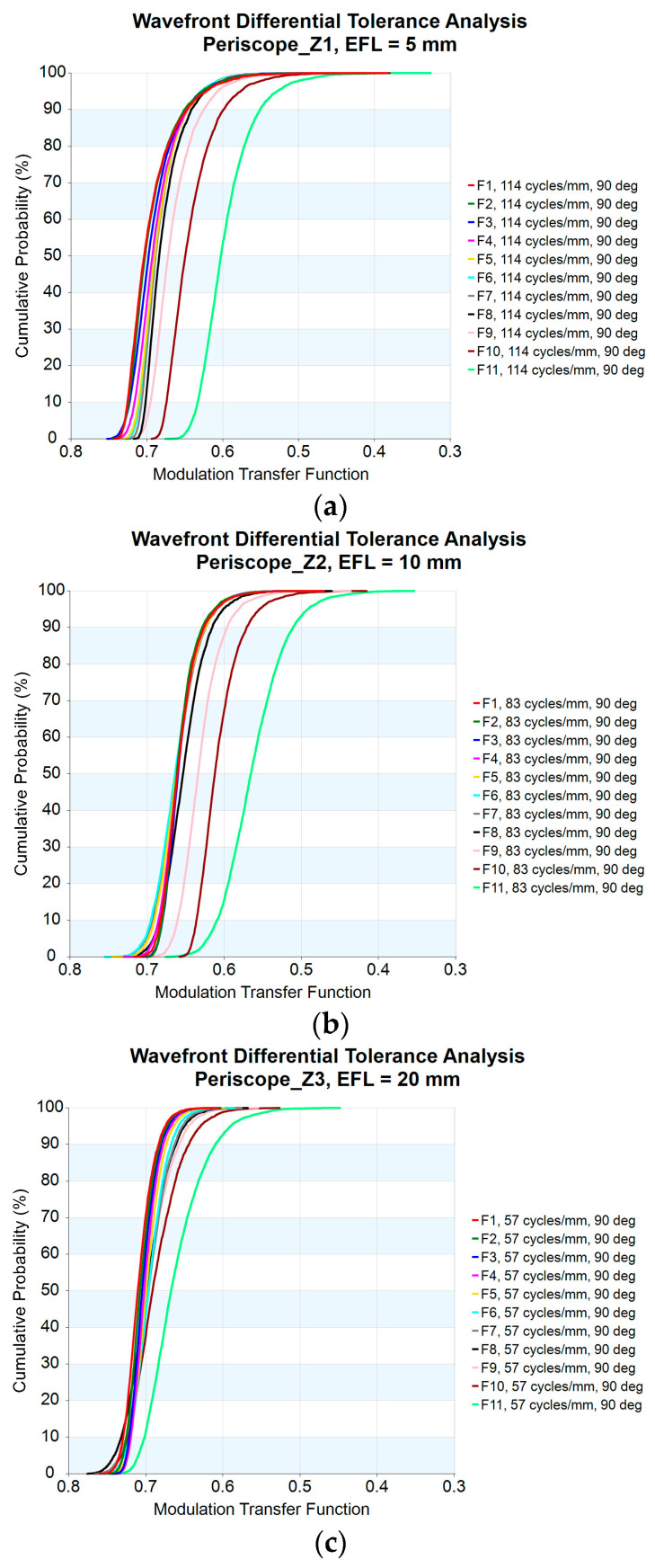

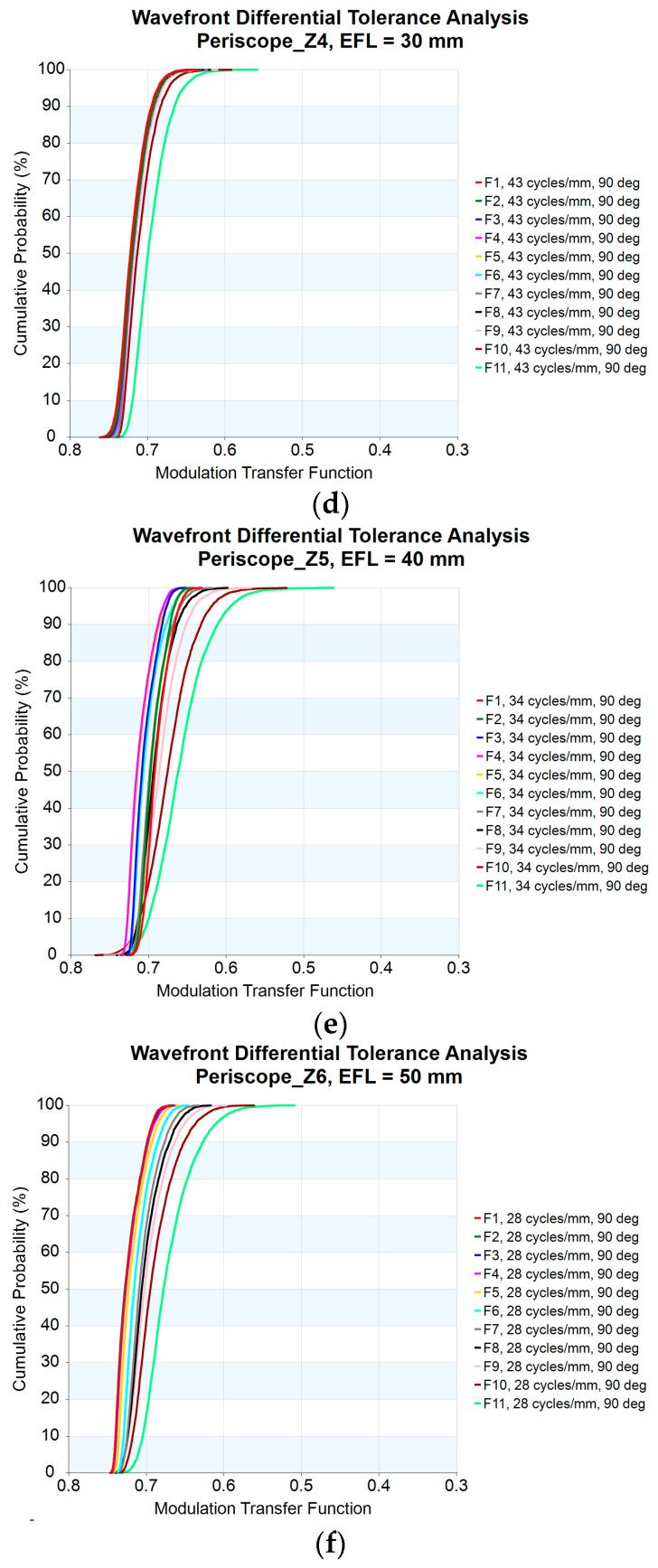
Cumulative probability versus MTF at (**a**) EFL = 5 mm; (**b**) EFL = 10 mm; (**c**) EFL = 20 mm; (**d**) EFL = 30 mm; (**e**) EFL = 40 mm; (**f**) EFL = 50 mm.

Figure 8a–f illustrate the cumulative probability versus MTF for the zoom lens obtained for focal lengths from 5 mm to 50 mm. It can be seen that all of the MTF values are greater than 0.45, indicating the resolution capability of this design. The minimum MTF is 0.48 for zoom 2, as shown in Figure 8c. The detailed data are shown in Table 6. Table 6 shows the image performance of the 10× periscope zoom lens. We show the different zoom positions from zoom 1 to zoom 6. Each zoom position can be compared with the relative field, MTF performance, and distortion. The MTF analysis includes design values and design plus tolerances at a spatial frequency of 43 lp/mm.

## 4. Conclusions

In order to miniaturize the high-magnification smartphone zoom lens, we start from geometric optical design to determine which optical constants will affect the lens thickness. The feasibility of actual lens production was also discussed. Aspheric molded glass lenses were selected for this design. To further decrease the camera depth, the circular aperture lens was changed to a rectangular aperture lens. This allows the removal of the unnecessary part of the circular aperture lens and simultaneously reduces the thickness of the lens. Finally, a tolerance analysis was performed to evaluate the manufacturing feasibility of this lens system. A 10× periscope zoom lens with an eight-megapixel image sensor was designed. The thickness of this lens was less than 6.5 mm, and the analytical results show that its optical performance meets the design requirements.

## Figures and Tables

**Table 1:** Fabrication data for the 10× periscope zoom lens.

Surface Number	Surface Type	Y Radius (mm)	Thickness (mm)	Glass	Refrac Mode	X-Full Aperture (mm)	Y-Full Aperture (mm)	Aperture Type	Tg (°C)
Object	Sphere	Infinity	Infinity		Refract				
1	Sphere	Infinity	3.2500	PSF68	Refract	10.1693	6.4494	Rectangular	428
2	Sphere	Infinity	−3.2500	PSF68	Reflect	8.3354	8.2941	Rectangular	428
3	Sphere	Infinity	−1.0000		Refract	7.8436	5.8563	Rectangular	
4	Asphere	49.2595	−1.0000	PSF68	Refract	7.5119	5.8173	Rectangular	428
5	Asphere	−29.0172	−0.2000		Refract	7.7113	6.1029	Rectangular	
6	Asphere	−15.5169	−2.3172	PLAF37	Refract	7.9711	6.2695	Rectangular	506
7	Asphere	9.9094	−0.3289 (D1)		Refract	7.9648	6.3113	Rectangular	
8	Asphere	4.8889	−0.8097	PLASF50	Refract	6.0262	4.1781	Rectangular	527
9	Asphere	−11.4143	−1.6901		Refract	4.5752	3.3608	Rectangular	
10	Asphere	27.3553	−1.0000	PLASF51	Refract	5.1843	5.1843	Circular	526
11	Asphere	−13.3212	−0.2462		Refract	5.6923	5.6923	Circular	
12	Asphere	−7.2740	−1.5648	EP8000	Refract	5.8167	5.8167	Circular	<200
13	Asphere	40.4822	−15.6656 (D2)		Refract	5.8199	5.8199	Circular	
Stop	Asphere	−5.2236	−5.0000	PMMAO	Refract	6.4078	6.4078	Circular	<200
15	Asphere	9.6013	−1.2086		Refract	5.5967	5.5967	Circular	
16	Asphere	21.4445	−2.4697	PSF68	Refract	4.5775	4.5775	Circular	428
17	Asphere	−14.2167	−2.2137 (D3)		Refract	4.3293	4.3293	Circular	
18	Asphere	18.0431	−1.9956	NFK58	Refract	4.8941	4.8941	Circular	445
19	Asphere	5.7075	−5.1262 (D4)		Refract	5.4892	5.4892	Circular	
20	Sphere	Infinity	−1.5590	NBK7	Refract	4.9177	4.9177	Circular	557
21	Sphere	Infinity	−0.1559		Refract	4.7960	4.7960	Circular	
22	Sphere	Infinity	−1.5590	NBK7	Refract	4.7775	4.7775	Circular	557
23	Sphere	Infinity	−2.3168		Refract	4.6562	4.6562	Circular	
Image	Sphere	Infinity	0.0000		Refract				

**Table 2:** Coefficient data for the aspheric surfaces.

Surface Number	K	A	B	C	D	E
4	53.58	8.77 × 10^−4^	6.04 × 10^−5^	−1.97 × 10^−6^	−3.65 × 10^−8^	2.16 × 10^−9^
5	−29.02	1.33 × 10^−3^	1.47 × 10^−5^	−5.27 × 10^−7^	−8.74 × 10^−8^	3.70 × 10^−9^
6	−30.68	5.92 × 10^−5^	−1.12 × 10^−5^	−9.58 × 10^−7^	5.11 × 10^−8^	−1.30 × 10^−9^
7	1.70	2.43 × 10^−5^	−1.23 × 10^−5^	−1.72 × 10^−6^	1.21 × 10^−7^	−3.64 × 10^−9^
8	−13.33	−1.00 × 10^−2^	6.69 × 10^−4^	−2.27 × 10^−5^	2.21 × 10^−7^	5.98 × 10^−9^
9	−6.63	−1.64 × 10^−2^	4.26 × 10^−4^	−2.04 × 10^−4^	1.01 × 10^−5^	1.34 × 10^−9^
10	−99.00	1.01 × 10^−2^	−2.02 × 10^−3^	1.42 × 10^−4^	−2.33 × 10^−6^	−2.89 × 10^−9^
11	−79.78	−6.81 × 10^−4^	2.30 × 10^−4^	2.60 × 10^−6^	1.72 × 10^−6^	1.82 × 10^−9^
12	−0.82	−3.37 × 10^−5^	4.89 × 10^−4^	−4.45 × 10^−5^	1.86 × 10^−6^	3.22 × 10^−9^
13	−99.00	1.54 × 10^−3^	−1.65 × 10^−4^	1.12 × 10^−5^	−7.51 × 10^−7^	−4.44 × 10^−9^
STOP	−0.86	−3.89 × 10^−5^	−1.29 × 10^−5^	2.44 × 10^−6^	−1.82 × 10^−7^	6.74 × 10^−9^
15	4.82	9.00 × 10^−4^	−2.29 × 10^−4^	9.69 × 10^−6^	−4.19 × 10^−7^	1.00 × 10^−8^
16	−14.63	2.91 × 10^−3^	−8.33 × 10^−5^	−3.81 × 10^−5^	3.09 × 10^−6^	−1.63 × 10^−10^
17	−9.80	−1.43 × 10^−4^	1.28 × 10^−4^	−6.39 × 10^−5^	4.30 × 10^−6^	−9.09 × 10^−14^
18	−20.06	6.35 × 10^−4^	5.17 × 10^−4^	−4.16 × 10^−5^	5.06 × 10^−6^	−2.79 × 10^−10^
19	0.84	−5.21 × 10^−4^	1.65 × 10^−4^	−2.04 × 10^−6^	6.22 × 10^−7^	3.51 × 10^−11^

**Table 3:** Zoom loci of the 10× periscope zoom lens.

	D1	D2	D3	D4
Zoom 1 EFL = 5 mm	−0.3289	−15.6656	−2.2137	−5.1262
Zoom 2 EFL = 10 mm	−2.7586	−8.7788	−3.2137	−8.5833
Zoom 3 EFL = 20 mm	−5.3418	−3.7490	−2.8277	−11.4159
Zoom 4 EFL = 30 mm	−6.3845	−0.4215	−3.3583	−13.1701
Zoom 5 EFL = 40 mm	−7.8023	−0.2000	−10.8663	−4.4659
Zoom 6 EFL = 50 mm	−8.0673	−0.1961	−14.871	−0.2000

**Table 4:** Diameter and position of entrance pupil at different zoom positions.

	Diameter of D_en_ (mm)	Position of D_en_ (mm)
Zoom 1	1.6667	11.4630
Zoom 2	2.5000	16.2022
Zoom 3	3.3333	24.2603
Zoom 4	3.7500	26.6939
Zoom 5	4.0000	37.6463
Zoom 6	4.1667	40.6413

**Table 5:** Tolerance values for the 10× periscope zoom lens.

Parameter	Tolerance Values
Delta Sag (DLS)	±λ/4
Delta Irregularity (IRR)	±1/50th λ
Delta Thickness (DLT)	±6.5 μm
Delta Index (DLN)	±0.0002
Delta Abbe Value (DLV)	±0.2%
Wedge (TIR)	±2.5 μm
Element Decenter (DIS)	±2.5 μm
Element Tilt (BTI)	$\pm 1^{\prime \prime }$
Mirror Tilt (TIL)	$\pm 1^{\prime \prime }$
Delta Sag (DLS)	±λ/4

**Table 6:** Image performance of the 10× periscope zoom lens.

Relative Field	Zoom 1				Zoom 2				Zoom 3			
	MTF			Distortion (%)	MTF			Distortion (%)	MTF			Distortion (%)
	Freq. (lp/mm)	Design	Design + Tolerance		Freq. (lp/mm)	Design	Design + Tolerance		Freq. (lp/mm)	Design	Design + Tolerance	
0.0, 0.0	114	0.7319	0.6235	0.0000	83	0.6826	0.6079	0.0000	57	0.7292	0.6709	0.0000
0.1, 0.1	114	0.7289	0.6266	0.0042	83	0.6828	0.6112	0.0164	57	0.7261	0.6694	0.0090
0.2, 0.2	114	0.7235	0.6251	−0.0079	83	0.6813	0.6096	0.0625	57	0.7231	0.6677	0.0360
0.3, 0.3	114	0.7169	0.6242	−0.0830	83	0.6828	0.6076	0.1298	57	0.7204	0.6646	0.0804
0.4, 0.4	114	0.7124	0.6251	−0.2393	83	0.6842	0.6046	0.2072	57	0.7183	0.6592	0.1412
0.5, 0.5	114	0.7122	0.6278	−0.4505	83	0.6862	0.6043	0.2835	57	0.7156	0.6509	0.2167
0.6, 0.6	114	0.7109	0.6276	−0.6807	83	0.6852	0.6022	0.3514	57	0.7160	0.6446	0.3042
0.7, 0.7	114	0.7072	0.6217	−0.9248	83	0.6782	0.5937	0.4087	57	0.7202	0.6411	0.4010
0.8, 0.8	114	0.7000	0.6037	−1.2096	83	0.6603	0.5756	0.4582	57	0.7221	0.6354	0.5050
0.9, 0.9	114	0.6814	0.5748	−1.5634	83	0.6410	0.5497	0.5053	57	0.7191	0.6218	0.6160
1.0, 1.0	114	0.6364	0.5240	−2.0000	83	0.6002	0.4807	0.5499	57	0.7030	0.5841	0.7372
**Relative Field**	**Zoom 4**				**Zoom 5**				**Zoom 6**			
	**MTF**			**Distortion** **(%)**	**MTF**			**Distortion** **(%)**	**MTF**			**Distortion** **(%)**
	**Freq.** **(lp/mm)**	**Design**	**Design + Tolerance**		**Freq.** **(lp/mm)**	**Design**	**Design + Tolerance**		**Freq.** **(lp/mm)**	**Design**	**Design + Tolerance**	
0.0, 0.0	43	0.7400	0.6840	0.0000	34	0.7057	0.6577	0.0000	28	0.7406	0.6909	0.0000
0.1, 0.1	43	0.7377	0.6819	0.0051	34	0.7107	0.6645	0.0031	28	0.7409	0.6911	−0.0060
0.2, 0.2	43	0.7375	0.6823	0.0207	34	0.7216	0.6754	0.0125	28	0.7411	0.6909	−0.0019
0.3, 0.3	43	0.7368	0.6822	0.0474	34	0.7282	0.6803	0.0287	28	0.7400	0.6892	−0.0028
0.4, 0.4	43	0.7379	0.6837	0.0858	34	0.7273	0.6773	0.0523	28	0.7365	0.6848	−0.0018
0.5, 0.5	43	0.7379	0.6839	0.1362	34	0.7204	0.6687	0.0838	28	0.7301	0.6766	0.0022
0.6, 0.6	43	0.7362	0.6818	0.1980	34	0.7126	0.6584	0.1236	28	0.7246	0.6670	0.0094
0.7, 0.7	43	0.7369	0.6809	0.2700	34	0.7120	0.6493	0.1715	28	0.7228	0.6589	0.0180
0.8, 0.8	43	0.7367	0.6778	0.3505	34	0.7097	0.6338	0.2272	28	0.7217	0.6498	0.0233
0.9, 0.9	43	0.7346	0.6705	0.4384	34	0.7049	0.6104	0.2896	28	0.7174	0.6357	0.0157
1.0, 1.0	43	0.7242	0.6505	0.5345	34	0.6980	0.5866	0.3580	28	0.7051	0.6088	−0.0207

## Data Availability

Not available.
